# Flotillin-2 is associated with breast cancer progression and poor survival outcomes

**DOI:** 10.1186/1479-5876-11-190

**Published:** 2013-08-15

**Authors:** Xi Wang, Qi Yang, Ling Guo, Xing-Hua Li, Xiao-Hui Zhao, Li-Bing Song, Huan-Xin Lin

**Affiliations:** 1 State Key Laboratory of Oncology in South China, Guangzhou 510060, PR China; 2 Department of Breast Oncology, Sun Yat-sen University Cancer Center, Guangzhou 510060, PR China; 3 Department of Nasopharyngeal Carcinoma, Sun Yat-sen University Cancer Center, Guangzhou 510060, PR China; 4 Department of Experimental Research, Sun Yat-sen University Cancer Center, Guangzhou 510060, PR China; 5 State Key Laboratory of Oncology in South China and Department of Radiation Oncology, Sun Yat-sen University Cancer Center, 651 Dongfeng Dong Road, Guangzhou 510060, PR China

**Keywords:** FLOT2, Breast cancer, Prognosis, Biomarker

## Abstract

**Background:**

Flotillin-2 (FLOT2) has been implicated in several signaling pathways in tumor cells. Our study aimed to investigate the expression pattern and clinicopathological significance of FLOT2 in patients with breast cancer.

**Methods:**

The expression level of FLOT2 in normal breast epithelial cells, breast cancer cell lines, and four breast cancer biopsies paired with adjacent noncancerous tissues were quantified using real-time RT-PCR and Western blotting. FLOT2 protein expression was analyzed in 171 archived paraffin-embedded breast cancer samples using immunohistochemistry (IHC). Statistical analyses were performed to evaluate the clinicopathological significance of FLOT2 expression.

**Results:**

FLOT2 was significantly upregulated in breast cancer cell lines and tissue samples compared with normal cells and adjacent noncancerous breast tissues, respectively. IHC analysis revealed high expression levels of FLOT2 in 82 of 171 (48.0%) breast cancer specimens. Statistical analysis revealed that FLOT2 expression was significantly correlated with clinical stage (P < 0.001), T classification (P < 0.001), M classification (P < 0.001), histological differentiation (P = 0.005) and ErbB2 expression (P = 0.003). Patients with higher levels of FLOT2 expression had a shorter overall survival duration than patients with lower FLOT2 expression levels. Multivariate analysis suggested that FLOT2 expression was an independent prognostic marker for survival in patients with breast cancer.

**Conclusions:**

The current results demonstrated that high FLOT2 protein expression was associated with poor outcomes in patients with breast cancer. FLOT2 could be used as a prognostic biomarker for breast cancer progression.

## Background

Breast cancer is the leading cause of cancer-related death in women worldwide, accounting for 23% of all new cancer cases and 14% of the total cancer deaths [1]. The etiological factors associated with breast cancer include both genetic and environmental factors. Altered expression levels of oncogenes and tumor suppressor genes have been found in breast cancer, but no specific signature of breast cancer gene expression has been reported to enable entirely individualized treatment strategies. In clinical practice, conventional pathological variables, including tumor size, nodal involvement and the depth of infiltration are extensively used to predict prognosis [2-5]. However, traditional pathological variables are not sufficiently reliable to predict clinical outcomes or to guide optimal treatment strategies. Recently, it has been reported that the molecular portraits revealed in gene expression patterns may lead to a deeper and more comprehensive understanding of breast cancer types [6]. Hence, the discovery of novel biomarkers involved in the diagnosis and progression of breast cancer is of great value in identifying high-risk patients who may benefit from more aggressive primary surgery, or adjuvant treatment following surgery, and in providing novel therapeutic targets.

FLOT2, a major protein on lipid rafts, is a highly conserved 47-kDa protein which was initially identified as a protein that was upregulated during axon regeneration after optic nerve lesion [7,8]. Given the role of lipid rafts in a number of cellular mechanisms that are dysregulated in tumor cells, such as altered protein signaling and trafficking, it is possible that abnormalities of FLOT2 protein contribute to the formation of cancer-specific cellular characteristics [9-11]. In 2000, Charles et al. identified a cluster of ER low-expression breast tumors that were partially characterized by the high level of expression of a specific subset of genes, including FLOT2 protein using cDNA microarrays [6]. FLOT2 overexpression has been reported to be associated with human melanoma progression and the development of metastasis in human head and neck squamous cell carcinomas [12,13]. Pust et al. reported that FLOT2, together with flot-1, Hsp90 and ErbB2, acted as a complex in breast cancer, and revealed that flotillins are implicated in the stabilization of ErbB2 at the plasma membrane [14]. They also showed that FLOT2 may serve as a potential predictor of prognosis in early-stage breast cancer by microarray analyses [14]. Shortly afterwards, FLOT2 was reported as a significant regulator of mammary tumor-derived lung metastasis [15]. However, the clinical significance of FLOT2 in breast cancer remains unclear.

In the present study, we found that the expression of FLOT2 was upregulated in breast cancer cells and surgical specimens of breast cancer. Moreover, the overexpression of FLOT2 in breast cancer is associated with the clinical stage, T and M classification, histological differentiation and ErbB-2 expression levels. Multivariate analysis revealed that FLOT2 might be an independent biomarker for the prediction of breast cancer prognosis. Taken together, our results suggest that FLOT2 plays a significant role in the development and progression of human breast cancer.

## Methods

### Cell lines

Primary normal mammary epithelial cells (NMEC) were established according to a previous report [16]. Breast cancer cell lines, including BT-549, MDA-MB-468, MDA-MB-453, Bcap37, MCF-7, T47D, ZR-75-1 and MDA-MB-231 were cultured in DMEM medium (Gibco, Grand Island, NY, USA) supplemented with 10% FBS (HyClone, Logan, UT, USA).

### Clinical samples and clinical staging system

This study was conducted on a total of 171 paraffin-embedded breast cancer samples, which were histopathologically and clinically diagnosed at the Sun Yat-sen University Cancer Center between 1998 and 2005. Clinical and clinicopathological classification and staging were determined according to the American Joint Committee on Cancer (AJCC) criteria. Patient consent was gained prior to the use of these clinical materials for research purposes, prior patients’ consents, and the protocol was approved from the Institutional Research Ethics Committee. Clinical information on the samples is summarized in Table 1 and Additional file 1: Table S2. The follow-up time of the primary breast cancer cohort ranged from 4 to 78 months, and the median follow-up time was 58 months. The percentages of tumor purity in sections adjacent to the regions used for RNA extraction were estimated during routine histopathological analyses.

**Table 1:** Clinicopathological characteristics of patient samples and expression of FLOT2 in breast cancer patients

	**Number of cases (%)**
**Sex**	
Male	0(0.0)
Female	171(100.0)
**Age (years)**	
≥47	85(49.7)
<47	86(50.3)
**Clinical stage**	
I	21(12.3)
II	92(53.8)
III	46(26.9)
IV	12(7.0)
**T classification**	
T_1_	35(20.5)
T_2_	89(52.0)
T_3_	35(20.5)
T_4_	12(7.0)
**N classification**	
N_0_	70(40.9)
N_1_	65(38.0)
N_2_	27(15.8)
N_3_	9(5.3)
**M classification**	
No	159(93.0)
Yes	12(7.0)
**Vital status (at follow-up)**	
alive	113(66.1)
Dead	58(33.9)
**Histological differentiation**	
Well	9(5.3)
Moderate	113(66.1)
Poor	49(28.7)
**Expression of FLOT2**	
Low expression	89(52.0)
High expression	82(48.0)
**Expression of ER**	
0	77(45.0)
1	82(48.0)
2	8(4.73)
3	4(2.3)
**Expression of PR**	
0	71(41.5)
1	80(46.8)
2	16(9.4)
3	3(1.8)
4	1(0.6)
**Expression of ErbB-2**	
0	42(24.6)
1	52(30.4)
2	38(22.2)
3	30(17.5)

### Real-time PCR (RT-PCR)

Total RNA samples from cell lines and primary tumor materials were extracted using Trizol reagent (Invitrogen, Carlsbad, CA, USA) according to the manufacturer’s instructions. The extracted RNA was pretreated with RNase-free DNase, and 2 μg RNA from each sample was used for cDNA synthesis primed with random hexamers. For the PCR amplification of FLOT2 cDNA, an initial amplification step using FLOT2–specific primers was performed with denaturation at 95°C for 10 min, followed by 28 denaturation cycles at 95°C for 60 s, primer annealing at 58°C for 30 s, and a primer extension phase at 72°C for 30 s. Upon the completion of the cycling steps, a final extension step at 72°C for 5 min was performed before the reaction mixture was stored at 4°C. Real-time PCR was then employed to determine the fold increase of FLOT2 mRNA in each of the primary breast tumors relative to the paired normal breast tissue taken from the same patient. The primers were designed using Primer Express v 2.0 software (Applied Biosystems) and are listed in Additional file 1: Table S1. Expression data were normalized to the geometric mean of Glyceraldehyde-3-phosphate dehydrogenase (GAPDH) to control the variability in expression levels, and all experiments were performed in triplicate.

### Western blotting

Cells at 70% to 80% confluence were washed twice with ice-cold phosphate-buffered saline (PBS) and lysed on ice in radio immunoprecipitation assay buffer (RIPA; Cell Signaling Technology, Danvers, MA) containing complete protease inhibitor cocktail (Roche Applied Sciences, Mannheim, Germany). Fresh tissue samples were ground to powder in liquid nitrogen and lysed with SDS-PAGE sample buffer. Equal protein samples (20 μg) were separated on 10.5% SDS polyacrylamide gels and transferred to PVDF membranes (Immobilon P, Millipore, Bedford, MA). Membranes were blocked with 5% fat-free milk in Tris-buffered saline containing 0.1% Tween-20 (TBST) for 1 h at room temperature. Membranes were incubated with anti-Flotillin-2 antibody (1:1000, Abcam, ab96507) overnight at 4°C, and then with horseradish peroxidase-conjugated goat anti-rabbit IgG (Santa Cruz Biotechnology, SC-2004). FLOT2 expression was detected using ECL prime Western blotting detection reagent (Amersham) according to the manufacturer’s instructions. P84 (Sigma, Saint Louis, MO) was used as a loading control.

### Immunohistochemical (IHC) analysis

Immunohistochemical analysis was performed to study altered protein expression in 171 human breast cancer tissues. Briefly, 4-μm-thick paraffin sections of the breast cancer tissue from the patient were deparaffinized with xylene and rehydrated. Antigenic retrieval was processed by submerging the sections into EDTA antigenic retrieval buffer and microwaving. The samples were then treated with 3% hydrogen peroxide in methanol to quench endogenous peroxidase activity, followed by incubation with 1% bovine serum albumin to block nonspecific binding. Sections were then incubated with anti-flotillin-2 rabbit polyclonal antibody (1:100, Abcam, ab96507) overnight at 4°C. Normal goat serum was used as a negative control. After washing, the tissue sections were then incubated with a biotinylated anti-rabbit secondary antibody (Abcam), followed by further incubation with streptavidin-horseradish peroxidase complex (Abcam). The tissue sections were immersed in 3-amino-9-ethyl carbazole and counterstained with 10% Mayer’s hematoxylin, dehydrated and mounted in Crystal Mount.

The degree of immunostaining of formalin-fixed, paraffin-embedded sections was evaluated independently by two observers who were blinded to the histopathological features and patient data of the samples. The scores given by the two independent investigators were averaged and based on both the proportion of positively-stained tumor cells and the intensity of staining. The proportion of tumor cells was scored as follows: 1 (<10% positive tumor cells), 2 (10-50% positive tumor cells), 3 (50-75% positive tumor cells), and 4 (>75% positive tumor cells). The intensity of staining was graded according to the following criteria: 0 (no staining); 1 (weak staining = light yellow), 2 (moderate staining = yellow brown), and 3 (strong staining = brown). The staining index was calculated as the product of the proportion of positive cells and the staining intensity score. Cut-off values for FLOT2 were chosen on the basis of a measure of heterogeneity using the log-rank test with respect to overall survival (OS). An optimal cut-off value was identified as follows: a staining index score of ≥6 was used to define tumors with high FLOT2 expression and ≤4 indicated low FLOT2 expression.

### Statistical analysis

The OS rate was the primary endpoint of this study, and the secondary endpoint was the disease-free survival (DFS) of patients with breast cancer. The OS was defined as the duration from the date of each patient’s random assignment to the date of death from any cause or the censoring of the patient at the date of the last follow-up. The DFS was defined as the time from randomization to local, regional, or distant treatment failure; occurrence of contralateral breast cancer; other second primary cancer; or death without evidence of breast or second primary cancer.

All statistical analyses were conducted using the SPSS 16.0 statistical software packages. The relationship between FLOT2 expression and clinicopathological characteristics was analyzed by the chi-square test and Fisher’s exact test. Bivariate correlations between study variables were calculated by Spearman’s rank correlation coefficients. Survival curves were plotted by the Kaplan–Meier method and compared using the log-rank test. Clinicopathological characteristics which were extensively used to predict prognosis in clinical practice were evaluated using univariate and multivariate Cox regression analyses. The type of Cox model chosen by us was forward method. In all cases, a P-value of less than 0.05 was considered statistically significant.

## Results

### FLOT2 is overexpressed in breast cancer cell lines

**Figure 1:**
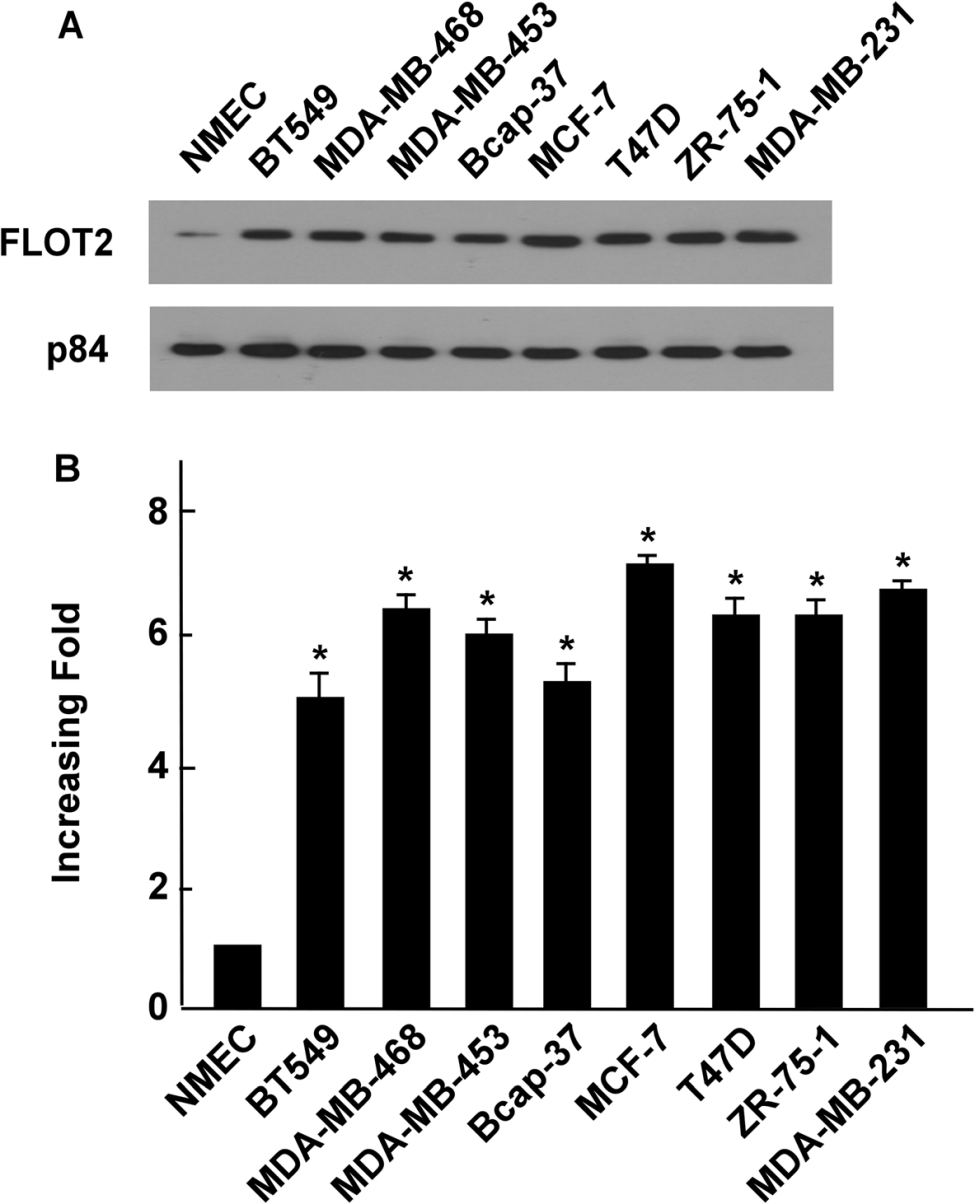
**Overexpression of FLOT2 mRNA and protein in breast cancer cell lines. (A and B)** Expression of FLOT2 mRNA and protein in breast cancer cell lines (BT-549, MDA-MB-468, MDA-MB-435, Bcap37, MCF-7, T47D, ZR-75-1 and MDA-MB-231) and NMEC were examined by Western blotting **(A)** and qPCR **(B)**. Expression levels were normalized against P84 and GAPDH respectively. Error bars represent the standard deviation of the mean (SD) calculated from three parallel experiments. *p < 0.05.

To evaluate the expression levels of FLOT2 protein and mRNA in breast cancer cell lines, we used Western blotting and real-time RT-PCR. The expression of FLOT2 mRNA and protein were determined for eight breast cancer cell lines (BT-549, MDA-MB-468, MDA-MB-453, Bcap37, MCF-7, T47D, ZR-75-1 and MDA-MB-231) and compared with FLOT2 expression in primary cultured normal mammary epithelial cells (NMEC). FLOT2 protein was highly expressed in breast cancer cell lines and only weakly expressed in NMEC (Figure 1A), and FLOT2 mRNA expression was expressed by at least 5-fold higher levels in breast cancer cell lines compared to NMEC (Figure 1B).

### FLOT2 is overexpressed in breast cancer tissues

**Figure 2:**
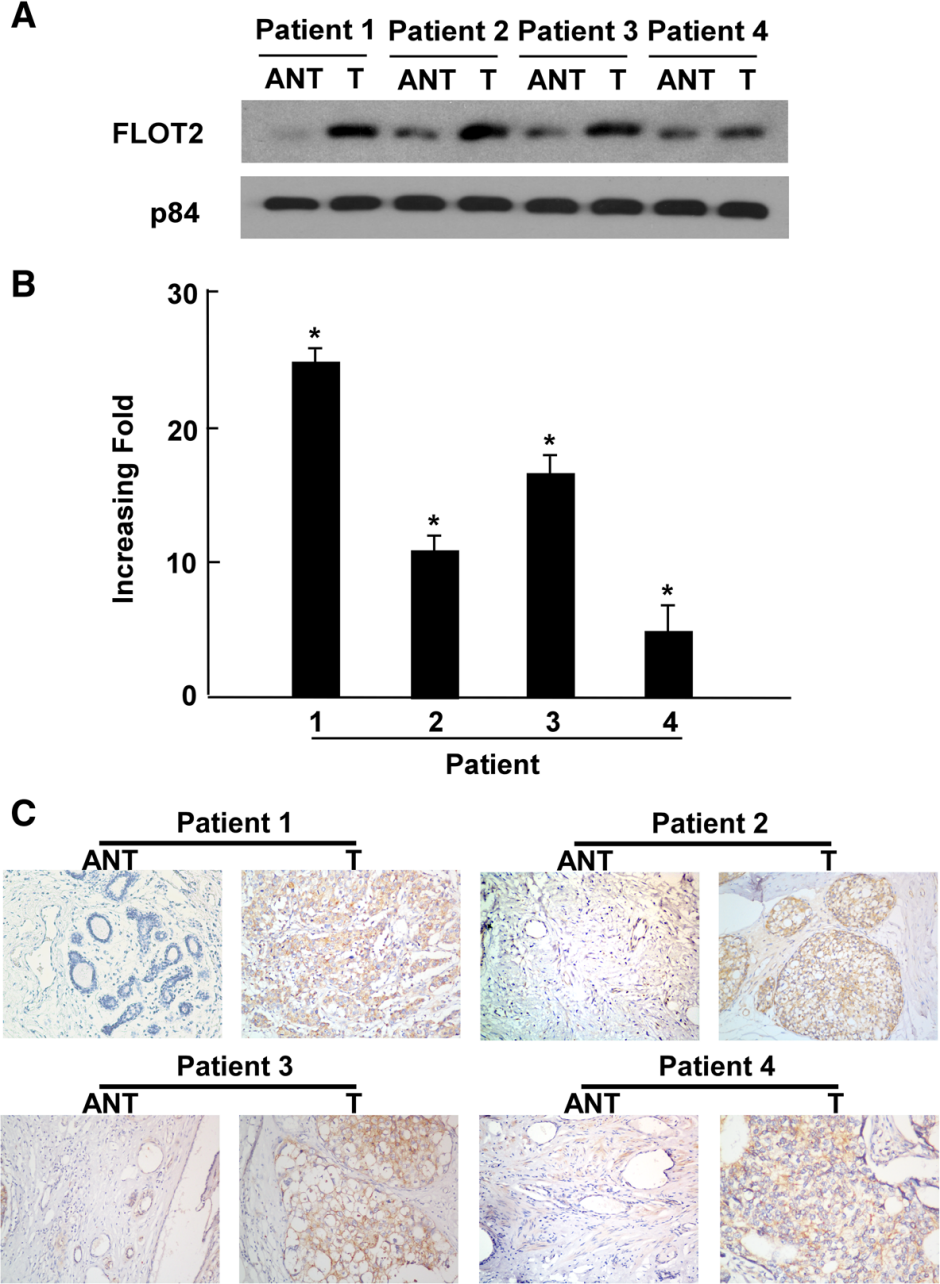
**Overexpression of FLOT2 mRNA and protein in breast cancer tissues. (A)** Representative images of Western blotting analyses of FLOT2 protein expression in four matched pairs of breast cancer (T) and adjacent noncancerous tissues (N). P84 was the loading control. **(B)** Average T/N ratios of FLOT2 mRNA expression in paired breast cancer (T) and adjacent noncancerous tissues (N) were quantified by qPCR and normalized against GAPDH. Error bars represent the standard deviation of the mean (SD) calculated from three parallel experiments. **(C)** Immunohistochemical assay of FLOT2 protein expression in four pairs of matched breast cancer tissues. *p < 0.05.

To determine whether FLOT2 is also high-expressed in the human breast cancer clinical samples, we performed RT-PCR and Western blotting analyses on four breast tumor samples (T) matched with adjacent noncancerous tissue samples (ANT). As illustrated in Figure 2B, FLOT2 mRNA was expressed at higher levels in all breast cancer tissues than in adjacent noncancerous tissues, with the differential expression level ranging from 5.1- to 24.4-fold. Consistent with this data, FLOT2 protein was also found to be upregulated in breast cancer tissues compared with the surrounding non-tumor regions (Figure 2A).

### FLOT2 overexpression is associated with breast cancer clinical features

**Figure 3:**
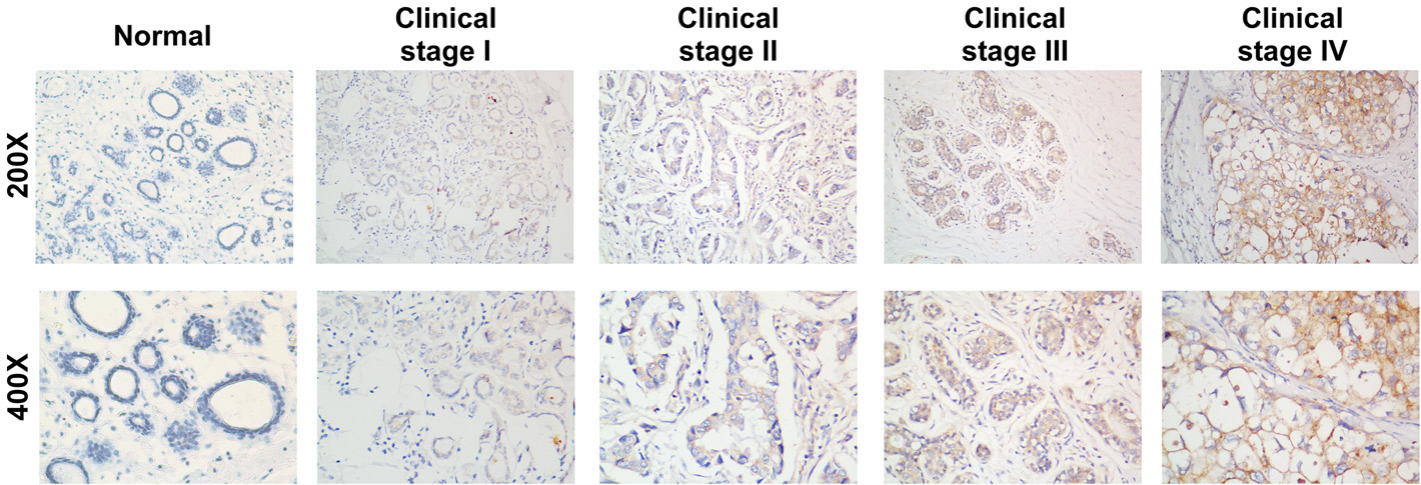
The expression of FLOT2 protein in breast cancer tissues from patients at different clinical stages.

We investigated the status of FLOT2 expression in 171 paraffin-embedded archived breast cancer tissues by immunohistochemical staining, including 21 stage I tumors, 92 stage II tumors, 46 stage III tumors and 12 stage IV tumors Among 171 samples, high FLOT2 protein expression was detected in 82 samples (48.0%) and weak or no staining was observed in 89 tumor samples (52.0%, Table 1). As shown in Figure 2C, no signals or only weak signals were detected in the adjacent non-cancerous tissues as well as normal breast tissues. In contrast, FLOT2 was highly expressed in breast cancer tissues. The subcellular location of FLOT2 was mainly at the plasma membrane (Figure 3). Furthermore, IHC staining showed that FLOT2 expression in breast cancer increased with advancing clinical stage (Figure 3). Taken together, these observations show that high levels of FLOT2 expression were associated with the clinical development of primary breast tumors.

We further analyzed the correlation between FLOT2 expression and the clinicopathological characteristics of patients. As summarized in Table 2, there were no significant correlations between the expression level of FLOT2 protein and patient age, N classification, estrogen receptor (ER) expression levels or progesterone receptor (PR) in patients with breast cancer. However, the FLOT2 expression level was markedly associated with clinical stage (P < 0.001), T classification (P < 0.001), M classification (P < 0.001), histological differentiation (P = 0.005) and ErbB2 expression levels (P = 0.003). These data were further confirmed by the results of Spearman correlation analyses that assessed the correlation between FLOT2 expression and clinicopathological features. As shown in Table 3, the correlation coefficients between FLOT2 expression and clinical stage, T classification, M classification, histological differentiation and ErbB2 expression levels were 0.306 (P < 0.001), 0.356 (P < 0.001), 0.286 (P < 0.001), 0.243 (P = 0.001) and 0.306 (P < 0.001), respectively. Taken as a whole, the expression of FLOT2 protein was positively correlated with clinical and pathological stage, T classification, M classification and ErbB-2 expression.

**Table 2:** Clinicopathological characteristics of patient samples and expression of FLOT2 in breast cancer patients and correlation between FLOT2 expression and clinicopathological characteristics of breast cancer patients

**Characteristics**		**Total (n = 171)**	**FLOT2**		**chi-square test *P*-value**	**Fisher’s exact test *P*-value**
			**Low expression 50.0%**	**High expression 50.0%**		
Age(y)	≥47	85	47(55.3)	38(44.7)	0.398	0.445
	<47	86	42(48.8)	44(51.2)		
Clinical stage	I	21	16(76.2)	5(23.8)	<0.001	<0.001
	II	92	53(57.6)	39(42.4)		
	III	46	20(43.5)	26(56.5)		
	IV	12	0(0.0)	12(100.0)		
T classification	T_1_	35	26(74.3)	9(25.7)	<0.001	<0.001
	T_2_	89	51(57.3)	38(42.7)		
	T_3_	35	11(31.4)	24(68.6)		
	T_4_	12	1(8.3)	11(91.7)		
N classification	N_0_	70	41(58.6)	29(41.4)	0.419	0.431
	N_1_	65	33(50.8)	32(49.2)		
	N_2_	27	11(40.7)	16(59.3)		
	N_3_	9	4(44.4)	5(55.6)		
M classification	Yes	12	0(0.0)	12(100.0)	<0.001	<0.001
	No	159	89(56.0)	70(44.0)		
Histological differentiation	Well	9	6(66.7)	3(33.3)	0.005	0.005
	Moderate	113	67(59.3)	46(40.7)		
	Poor	49	16(32.7)	33(67.3)		
Expression of ER	0	77	38(49.4)	39(50.6)	0.155	0.165
	1	82	41(50.0)	41(50.0)		
	2	8	7(87.5)	1(12.5)		
	3	4	3(75.0)	1(25.0)		
Expression of PR	0	71	35(49.3)	36(50.7)	0.498	0.527
	1	80	41(51.2)	39(48.8)		
	2	16	11(68.8)	5(31.2)		
	3	3	1(33.3)	2(66.7)		
	4	1	1(100.0)	0(0.0)		
Expression of ErbB-2	0	42	30(71.4)	12 (28.6)	0.003	0.002
	1	52	30(57.7)	22(42.3)		
	2	38	18(47.4)	20(52.6)		
	3	30	9(30.0)	21(70.0)		
	4	9	2(22.2)	7(77.8)		

**Table 3:** Spearman correlation analysis between FLOT2 and clinical pathologic factors

**Variables**	**FLOT2 expression level**	
	**Spearman correlation**	***p-Value***
Clinical staging	0.306	<0.001
T classification	0.356	<0.001
M classification	0.286	<0.001
Histological differentiation	0.243	0.001
ErbB-2	0.306	<0.001

### Association between FLOT2 expression and patient survival

**Figure 4:**
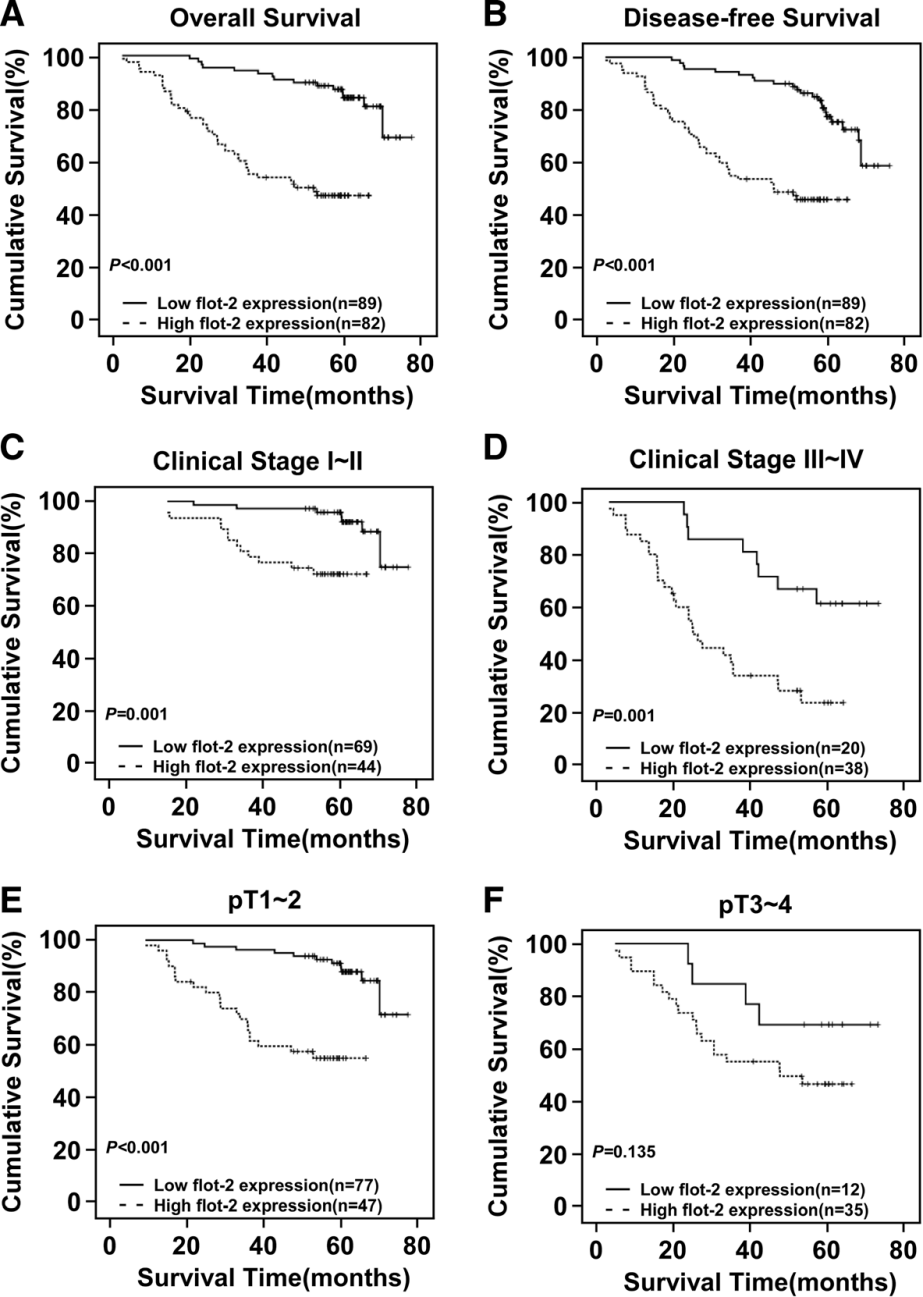
**Kaplan-Meier curves with univariate analysis (log-rank). (A and B)** OS **(A)** and 5-year DFS **(B)** rates for cases with high FLOT2 expression versus those for cases with low FLOT2 expression levels in all patients. **(C)** OS rate for early clinical stage cases (stage I/II) with high FLOT2 expression versus those for cases with low FLOT2 expression levels. **(D)** OS rate for late stage cases (stage III/IV) with high FLOT2 expression levels versus cases with low FLOT2 expression. **(E)** OS rate for cases with high FLOT2 expression versus cases with low FLOT2 expression in patients with T1-T2 grade tumors. **(F)** OS rate for cases with high FLOT2 expression versus that for cases with low FLOT2 expression in patients with T3-T4 grade tumors.

Patient survival analysis showed a clear negative correlation between the level of FLOT2 protein expression and both the OS and 5-year DFS of patients with breast cancer (both P < 0.001, Figures 4A, B). The cumulative OS and DFS rates for patients with high levels of FLOT2 expression were found to be 47.7% and 45.8%, respectively, whereas the rates were 87.3% and 83.7%, respectively, for patients with low or no FLOT2 expression. In addition, Cox regression revealed that FLOT2 expression, clinical stage, ErbB2 expression and histological differentiation were independent prognostic factors for poor OS outcomes (Table 4).

**Table 4:** Univariate and multivariate analyses of various prognostic parameters in patients with breast cancer Cox-regression analysis (overall survival was modelled)

	**Univariate analysis**			**Multivariate analysis**		
	**No. patients**	***p***	**Regression coefficient (SE)**	***p***	**Relative risk**	**95% confidence interval**
**FLOT2**				0.002		
Low expression	89	<0.001	1.209(0.172)		Ref	
High expression	82			0.002	4.492	1.740-11.598
**Clinical stage**				<0.001		
I	21				Ref	
II	92	<0.001	1.618(0.314)	0.824	1.190	0.256-5.522
III	46			0.329	2.236	0.444-11.264
IV	12			0.014	8.594	1.545-47.790
**ErbB-2**				0.06		
0	42				Ref	
1	52	<0.001	0.376(0.106)	0.272	0.602	0.244-1.489
2	38			0.237	1.625	0.727-3.629
3	30			0.164	1.811	0.785-4.178
4	9			0.253	2.005	0.608-6.609
**Histological differentiation**				0.005		
Well	9	<0.001	1.283(0.262)		Ref	
Moderate	113			0.783	0.810	0.181-3.631
Poor	49			0.327	2.105	0.475-9.330

Moreover, we analyzed the prognostic value of FLOT2 expression in selective patient subgroups stratified according to tumor grade and T classification (pT), respectively. The expression of FLOT2 was strongly associated with the OS duration of patients with both early-stage tumors (Stage I or II, log-rank test, P = 0.001) and late-stage tumors (Stage III and IV, log-rank test, P = 0.001) (Figures 4C and D). However, when it was examined according to T classification, the impact on outcome associated with the positive expression of FLOT2 continued to be more favorable only in the pT1–2 subset (Figure 4E, log-rank test, P < 0.001) but not in the pT3-4 subset (Figure 4F, log-rank test, P = 0.135).

## Discussion

In this report, we present new evidence that the overexpression of FLOT2 is associated with poor prognosis in breast cancer patients with both early- and late-stage disease. Our results clearly showed that the upregulation of FLOT2 occurred at both the levels of mRNA and protein in breast cancer cell lines compared to NMEC. Paired breast cancer lesions and adjacent noncancerous tissues were found to have different expression levels of FLOT2, with cancer lesions displaying relatively higher expression levels of FLOT2. IHC analyses indicated that FLOT2 was highly expressed in breast cancer tissues, which was significantly correlated with the clinical stage of the disease and unfavorable survival durations. Our study used biopsy material from both early-stage (stage I/II) and late-stage (stage III/IV) disease to analyse the correlation between FLOT2 expression and survival times, whereas a similar study by Pust et al. only used tissue from early-stage (stage I/II) disease [14]. Multivariate analysis revealed that FLOT2 expression might be an independent prognostic indicator of survival in breast cancer patients. Our results suggest the important role of FLOT2 protein in the prognosis of patients with breast cancer.

FLOT2 has been implicated in several signaling pathways in tumor cells. Xenograft experiments revealed that FLOT2 overexpression facilitated the transformation of the non-metastatic melanoma cell line (SB2) into a highly tumorigenic metastatic cell line [12]. FLOT2 might promote PAR-1-induced constitutive signaling through the BRAF-MAPK-ERK pathway implicated in melanoma progression [17]. The FLOT2-mediated stabilization of ErbB2 has been associated with tumorigenesis in stage I/II breast cancer tissues, which is reflected by reduced p-ErbB2 and p-Akt levels and hyperactivity of the PI3K/AKT/mTOR pathway in breast tumors [14,18]. Data from a very recent publication of the genetic ablation of FLOT2 in a well-established mouse model of mammary tumorigenesis and metastasis indicated that FLOT2 predicts mammary tumor-derived lung metastasis. We therefore hypothesize that FLOT2 may affect the development and progression of breast cancer through modulating certain signaling pathways, which may open new avenues into the treatment of breast cancer. As many pathways have been reported to be hyperactive in breast tumors, the protein kinases located along the pathway regulated by FLOT2 represent attractive drug targets for breast cancer therapies. However, further studies are required to obtain a detailed picture of FLOT2-related signaling pathways in breast cancer development.

We further analyzed the relationship between the expression of FLOT2 and clinical characteristics of patients affected by breast cancer. There was a significant correlation between FLOT2 expression and the clinical stage, T classification, M classification, histological differentiation and ErbB2 expression levels, which strongly suggested that the overexpression of FLOT2 would be useful as an independent marker for the identification of subsets of breast cancer patients with more aggressive disease. Moreover, patients in the high FLOT2 expression group had a 47.7% cumulative 5-year survival rate, which was significantly lower than that of patients with low FLOT2 expression levels (87.3%). This finding indicates the possibility of using high expression levels of FLOT2 as a predictor for prognosis and survival.

In conclusion, this is the first study to highlight the clinical significance of FLOT2 in breast cancer. Higher FLOT2 expression levels are a significant prognostic marker of poor survival in breast cancer patients. A comprehensive analysis of the molecular mechanism of FLOT2 involvement in the development and progression of breast cancer is eagerly awaited. Further investigation is also needed to determine whether FLOT2 could be identified as a target for novel therapeutics against breast cancer.

## Conclusions

In this study, we found that overexpression of FLOT2 is associated with poor prognosis and reduced survival of patients with both early-and late-stage disease. Multivariate analysis showed that FLOT2 protein levels could be used as an independent prognostic predictor for breast cancer patients. Therefore, testing the FLOT2 protein level may be useful for stratifying patients for novel therapeutic strategies and establishing rational treatment selection criteria for patients with breast cancer.

## Competing interests

The authors declare that they have no competing interests.

## Authors’ contributions

XW collected the tissue specimens and patient information, and carried out the statistical analyses. QY carried out the Western blotting, and drafted the manuscript. LG participated in collecting patient information and editing of the manuscript. XL carried out Immunohistochemical (IHC) analysis. XZ carried out RNA extraction and real-time PCR. LS participated in conceiving the study and guiding the editing of the manuscript. HL conceived the study, wrote and guided the editing of the manuscript. All authors read and approved the final manuscript.

## Supplementary Material

Additional file 1: Table S1 Primer sequences used real-time quantitative reverse transcription-PCR (5' to 3’). **Table S2.** Adjuvant treatments categories of the patients. Click here for file
