# Supplementary material for: Flotillin-2 is associated with breast cancer progression and poor survival outcomes
Source: J Transl Med. 2013 Aug 15;11:190. doi: 10.1186/1479-5876-11-190 (PMC3765773; doi:10.1186/1479-5876-11-190)
Supplement: Additional file 1: Table S1 — Primer sequences used real-time quantitative reverse transcription-PCR (5' to 3’). Table S2. Adjuvant treatments categories of the patients. [file 1479-5876-11-190-S1.pdf]

Supplementary Information

Supplementary Table 1. Primer sequences used real-time quantitative reverse transcription-PCR (5' to 3')

Gene	Forward primer	Reverse primer
FLOT2	TTGCTGACTCTAAGCGAGCC	TCCACGGCAATCTGTTTCTTG
GAPDH	GACTCATGACCACAGTCCATGC	AGAGGCAGGGATGATGTTCTG

Supplementary Table 2. Adjuvant treatments categories of the patients

	Number of cases (%)	
	+	−
chemotherapy	143(83.6)	28(16.4)
endocrine	89(52.0)	82(48.0)
trastuzumab	17(10.0)	154(90.0)
